# Hypotensive Resuscitation among Trauma Patients

**DOI:** 10.1155/2016/8901938

**Published:** 2016-08-09

**Authors:** Matthew M. Carrick, Jan Leonard, Denetta S. Slone, Charles W. Mains, David Bar-Or

**Affiliations:** ^1^Trauma Services Department, The Medical Center of Plano, 3901 W. 15th Street, Plano, TX 75075, USA; ^2^Department of Trauma Research, Medical Center of Plano, 3901 W. 15th Street, Plano, TX 75075, USA; ^3^Department of Trauma Research, Swedish Medical Center, 501 E. Hampden Avenue, Englewood, CO 80113, USA; ^4^Department of Trauma Research, St. Anthony Hospital, 11600 W. 2nd Place, Lakewood, CO 80228, USA; ^5^Department of Trauma Research, Penrose Hospital, 2222 N. Nevada Avenue, Colorado Springs, CO 80907, USA; ^6^Trauma Services Department, Swedish Medical Center, 501 E. Hampden Avenue, Englewood, CO 80113, USA; ^7^Rocky Vista University, 8401 S. Chambers Road, Parker, CO 80134, USA; ^8^Trauma Services Department, St. Anthony Hospital, 11600 W. 2nd Place, Lakewood, CO 80228, USA

## Abstract

Hemorrhagic shock is a principal cause of death among trauma patients within the first 24 hours after injury. Optimal fluid resuscitation strategies have been examined for nearly a century, more recently with several randomized controlled trials. Hypotensive resuscitation, also called permissive hypotension, is a resuscitation strategy that uses limited fluids and blood products during the early stages of treatment for hemorrhagic shock. A lower-than-normal blood pressure is maintained until operative control of the bleeding can occur. The randomized controlled trials examining restricted fluid resuscitation have demonstrated that aggressive fluid resuscitation in the prehospital and hospital setting leads to more complications than hypotensive resuscitation, with disparate findings on the survival benefit. Since the populations studied in each randomized controlled trial are slightly different, as is the timing of intervention and targeted vitals, there is still a need for a large, multicenter trial that can examine the benefit of hypotensive resuscitation in both blunt and penetrating trauma patients.

## 1. Introduction

In the United States, trauma is the leading cause of death for persons between the ages of 1 and 44 years and the fifth leading cause of death overall [1]. Globally, over 5 million people die of injuries each year, accounting for 9% of the world's deaths [2]. Hemorrhagic shock is a principal cause of death among trauma patients accounting for approximately 30–40% of deaths within the first 24 hours after injury [3]. Vascular disruption, blood pressure, volume resuscitation, and the time between injury and hemostasis are all factors that contribute to the magnitude of hemorrhage [4].

Over the past 30 years, there has been a renewed interest in research focusing on the optimal resuscitation strategies for trauma patients, specifically those with uncontrolled hemorrhage, in hopes of decreasing mortality from hemorrhagic shock. This review focuses on hypotensive resuscitation, also called permissive hypotension. This resuscitation strategy uses limited fluids and blood products during the early stages of treatment for hemorrhagic shock in order to maintain a lower-than-normal blood pressure until operative control of the bleeding can occur. The goal is to limit additional bleeding due to “popping the clot” while still providing oxygen to the tissues [4, 5]. The use of restricted fluids has been shown to improve outcomes in animal models [6]; until recently, few randomized controlled trials on hypotensive resuscitation existed in trauma patients [7–11].

## 2. History of Fluid Administration and Resuscitation Strategies

The idea of hypotensive resuscitation was introduced as far back as 1918 when Walter Cannon reported observations from World War I in “The Preventive Treatment of Wound Shock.” Cannon stated that

*“Injection of fluid that will increase blood pressure has dangers in itself. Hemorrhage in a case of shock may not have occurred to a marked degree because blood pressure has been too low and the flow too scant to overcome the obstacle offered by a clot. If the pressure is raised before the surgeon is ready to check any bleeding that may take place, blood that is sorely needed may be lost [12].”*

After World War II, Beecher reiterated that when blood transfusions or surgery are inaccessible, rapid plasma administration is not desirable; the plasma may elevate the blood pressure to a degree where increased bleeding occurs. Instead, the time for fluid administration is when surgery is available [13].

However, animal research in the 1950s and 1960s found value in supplementing the replacement of lost blood with both whole blood and a balanced salt solution [14, 15]. A suggested treatment algorithm for trauma patients presenting to the emergency room in hemorrhagic shock proposed using a large-gauge needle or catheter to immediately infuse 1000 to 2000 mL of crystalloid (i.e., lactated Ringer's solution or 5% dextrose in water) over the course of approximately 45 minutes. While the fluids were being infused, the patient's blood was typed and cross-matched so that whole blood could later be administered [16–18]. The use of crystalloids for initial resuscitation was conveyed as beneficial because it raised the blood pressure to a normal level and reduced the amount of whole blood needed by patients [16, 17]. Despite calls for moderation [19], when attempting to reverse hemorrhagic shock, it became routine for surgeons and emergency medical personnel to practice expedient hemorrhage control and use high-volume fluid resuscitation strategies to replace lost blood [20].

## 3. Complications Attributable to Aggressive Fluid Administration

A critique of large volume fluid resuscitation is that the administration of excessive fluid contributes to and exacerbates the lethal triad of hypothermia, acidosis, and coagulopathy, thereby increasing bleeding and mortality [21–24].

Hypothermia and acidosis inhibit the generation of thrombin and the availability of fibrinogen leading to increased or continued bleeding [25]. Trauma patients are already at an increased risk of hypothermia due to the potential to lose body heat while at the scene of the injury, through decreased heat production attributable to hemorrhagic shock and diminished oxygen consumption or from open cavities during operative procedures [4, 24]. Infusing 2 liters of 25-degree Celsius saline or lactated Ringer's solution decreases a 70-kilogram patient's body temperature by up to one-third of a degree Celsius [26]. Infusing warm rather than room temperature fluids is an important component of fluid resuscitation. While fluid warming prevents additional heat loss, additional measures such as warming blankets and heated trauma bays and operating rooms need to be incorporated as well [4, 25, 27].

Coagulopathy, the inability for the blood to clot, is present on admission to the hospital in approximately 25% of trauma patients [28]. Medical interventions, like giving large quantities of fluid, are thought to aggravate coagulopathy through multiple pathways. When fluids are given, the increased blood pressure may cause the thrombus that was forming to become dislodged, thus contributing to continued bleeding [29]. Large volumes of fluid can also dilute the coagulation products that are within the blood, especially if the fluids used to replace lost blood do not contain platelets or other clotting factors [4, 21, 29].

Lastly, large volume fluid resuscitation has been associated with increased mortality [7, 10, 30] and other comorbid conditions [21, 29]. Among a blunt trauma population, Kasotakis et al. found large volume crystalloid fluid resuscitation was not associated with increased mortality, but it was associated with many complications including acute lung injury/adult respiratory distress syndrome, multiple organ failure, abdominal compartment syndrome, and surgical site infections. Additionally, time on the ventilator, intensive care length of stay, and the overall hospital length of stay were increased in patients with higher volumes of crystalloid resuscitation [31].

## 4. Aggressive versus Hypotensive Fluid Administration

Critics of aggressive fluid resuscitation cite the abovementioned complications, while those skeptic of hypotensive resuscitation are concerned with the potential harmful effects of decreased oxygen delivery to the various tissues of the body, which could result in inadequate perfusion and subsequent organ failure [32]. Since the early 1990s, a handful of randomized controlled trials have been conducted in trauma patients either in the prehospital or in-hospital and intraoperative setting [7–11].

## 5. Hypotensive Resuscitation Randomized Controlled Trials

Five randomized controlled trials have been conducted over the past 30 years to explore the differences between restricted and aggressive fluid administration. None of these studies were conducted in pregnant women.

Bickell et al. conducted the landmark prospective, randomized study examining immediate versus delayed fluid resuscitation among penetrating trauma patients, which found greater survival in patients treated with delayed resuscitation compared to immediate resuscitation. Patients ≥16 years old who suffered a penetrating torso injury and had a systolic blood pressure (SBP) of 90 mmHg or lower were included in this trial. The intervention was conducted in the prehospital setting. The immediately resuscitated group received rapid infusion of Ringer's solution while being transferred to the hospital; fluid administration was continued at the hospital if the SBP was below 100 mmHg. The delayed resuscitation group did not receive fluids in-route or when initially arriving at the hospital. With the exception of the fluid administration described, both groups were otherwise treated following the same protocol. Both groups could receive fluids once under anesthesia to maintain prespecified vitals in the operating room. Over approximately 3 years, 598 patients completed the study, of which 51.7% were immediately resuscitated and 48.3% received delayed resuscitation. In addition to increased survival in the delayed versus immediate resuscitation group (70% versus 62%, *p* = 0.04), the delayed group had a shorter hospital length of stay. No differences were found between groups in the rates of respiratory distress syndrome, sepsis, acute renal failure, coagulopathy, wound infection, or pneumonia [7].

Turner et al. did not randomize trauma patients but rather paramedics, in order to examine the effect of the intravenous fluid administration policy that was being used to treat trauma patients in the prehospital setting. In this study, paramedics followed one of two protocols and crossed over to the opposite protocol halfway through the study. In Protocol A, intravenous fluids were administered at the scene to all adult trauma patients who would typically receive intravenous fluids. In Protocol B, fluids were withheld from the patients until arrival at the hospital or were given in a delayed manner when the time to the hospital was scheduled to take over one hour. Over 17 months, 1309 patients were randomized into the study; 699 (53.4%) followed Protocol A and 610 (46.6%) were treated by Protocol B. There was no difference in mortality at 6 months after the injury; 73 (10.4%) people in Protocol A died and 60 (9.8%) people following Protocol B died. There was no difference between the two groups in the number of patients with at least one of the following complications: adult respiratory distress syndrome, sepsis, acute renal failure, coagulopathy, wound infection, pneumonia, fat embolism, or pulmonary embolism. While the study included all trauma patients, the population was primarily made up of blunt injuries; only 24 (1.8%) penetrating injuries occurred in the study. Finally, the protocol compliance was poor. Only 31% of patients in Protocol A actually received intravenous fluids [8].

The third randomized controlled trial by Dutton et al. studied blunt and penetrating trauma patients with ongoing hemorrhage and at least one SBP < 90 mmHg. At the hospital, patients were prospectively randomized to a conventional group that received fluid administration to reach SBP > 100 mmHg while those in the low group received fluid to target SBP of 70 mm  Hg. A combination of crystalloid or blood products was used. For both groups, fluid administration ceased after reaching the target blood pressure. During a 20-month enrollment period, 110 patients were enrolled in the study with half of the patients (*n* = 55) in each group. Interestingly, despite a target SBP of 70 mmHg and greater than 100 mmHg, the average SBP during bleeding was 100 ± 17 and 114 ± 12 (*p* < 0.001), respectively. No difference was found in the in-hospital mortality when trauma patients with hemorrhage were resuscitated at the hospital with conventional (target SBP > 100 mmHg) and low (target SBP: 70 mmHg) strategies. Perhaps the higher-than-intended SBP in the low group contributed to the equal number of deaths in each group (*n* = 4 per group). Among the four patients in the conventional group who died, two patients died while actively hemorrhaging and the other two died of multiple organ failure. In the low strategy resuscitation group, 3 patients died while actively hemorrhaging and one died from fulminant hepatic failure after suffering a grade V blunt liver injury that required a near total resection and embolization. This study only examined overall in-hospital mortality and did not look at mortality at different time points or additional complications [9].

An out-of-hospital, prospective, randomized pilot trial conducted by Schrieber et al. assessed the feasibility and safety of hypotensive resuscitation for the early resuscitation of patients with traumatic shock due to blunt or penetrating trauma. Trauma patients who were older than 15 years with an out-of-hospital SBP of ≤90 mmHg were studied. Patients randomized to standard resuscitation received an initial 2 L bolus of fluid with additional fluid given to maintain SBP of 110 mmHg. In the controlled resuscitation group, a 250 mL bolus of fluid was only given to maintain SBP of 70 mmHg. The early resuscitation efforts continued until two hours into the hospital stay or until the hemorrhage was under control, whichever came first. One hundred ninety-two patients were randomized; 95 (49.5%) received standard resuscitation and 97 (50.5%) underwent controlled resuscitation. Patients who were randomized to controlled resuscitation received approximately 1 L less of fluid during the early resuscitation period than those with standard resuscitation. While the difference in 24-hour mortality (5.2% versus 14.7%) and in-hospital mortality (8.4% versus 16.5%) was not statistically significant between groups, it did favor controlled resuscitation. Interestingly, when injury type was examined, the blunt trauma subgroup that received controlled resuscitation had decreased mortality (3.2% versus 17.7%; adjusted odds ratio: 0.17; 95% confidence interval: 0.03–0.92). No statistically significant differences were observed in the need for major surgical procedures, renal function, ICU-free days, or ventilator-free days. Schreiber et al. concluded that hypotensive resuscitation is feasible and safe for the initial resuscitation of trauma patients [10].

Most recently, Carrick et al. published the results of their study that included younger patients (14–45 years) with penetrating injury who underwent a laparotomy or thoracotomy to control hemorrhaging; the hypotensive resuscitation strategy was implemented intraoperatively. The experimental group had a targeted minimum mean arterial pressure (MAP) for resuscitation of 50 mmHg (LMAP) and the control group targeted a minimum MAP of 65 mmHg (HMAP). The trial enrolled 168 patients (86 LMAP, 82 HMAP). In the end, this trial was terminated early due to temporal changes in processes of care, lack of equipoise, slow accrual, and futility and therefore was underpowered and unable to demonstrate an improvement in 30-day mortality. While there was a 5% difference in mortality favoring the hypotensive group, this was not statistically significant. Secondary complications were examined; no differences were noted between the two study groups in acute myocardial infarction, stroke, renal failure, hypotension, coagulopathy, thrombocytopenia, anemia, and infection (*p* > 0.05 for all). Acute renal injury was significantly higher in the HMAP group (13% versus 30%,  *p* = 0.01). Like in the Dutton et al. study [9], although the LMAP and HMAP study groups had different target intraoperative MAPs, no difference was observed in the mean MAPs recorded intraoperatively (65.5 ± 11.6 mmHg versus 69.1 ± 13.8 mmHg, *p* = 0.07). While the mean MAP for each patient was not statistically different between groups, when analyzing each intraoperative mean arterial pressure recorded approximately every 20 seconds, the amount of time spent below the target MAP was significantly less for the LMAP group compared to the HMAP group (12.6% versus 35.2%, *p* < 0.001). This suggests that it is not as difficult for a patient to independently maintain an intraoperative MAP above 50 mmHg as it is to maintain one above 65 mmHg [11].

In the brief descriptions of the randomized controlled trials above and in Tables 1 and 2, it is evident that each study takes place in a slightly different location, with differing populations and various fluid administration protocols. Three of the studies were conducted in the prehospital setting [7, 8, 10] and two were conducted upon arrival to the hospital [9] or intraoperatively [11]. Two studies were conducted within only penetrating trauma populations [7, 11], whereas three examined blunt or penetrating traumas [8–10]. Three studies excluded patients with a known or suspected head injury [9–11]. The studies by Turner et al. and Schreiber et al. specifically excluded burn patients [8, 10]. To be included in the Bickell et al. and Carrick et al. studies, a patient had to have an operative procedure performed [7, 11]. Finally, the fluid administration process for each study varied; Bickell et al. and Turner et al. gave a specified amount of fluid regardless of vitals [7, 8], Dutton et al. and Schreiber et al. gave fluid to a targeted SBP [9, 10], and Carrick et al. administered fluid to a targeted MAP [11].

Though the outcomes of each study differed, none of the studies found restricted fluid resuscitation to be harmful or worse than large volume fluid resuscitation (Tables 1 and 2). Bickell et al.'s prehospital study was the only study that found significantly increased survival in patients who received delayed fluid resuscitation compared to those in the immediate resuscitation group (70% versus 62%, *p* = 0.04) [7]. The other four studies did not find a significant difference in mortality. However, mortality at 24 hours (5.2% versus 14.7%) [10], in-hospital mortality (8.4% versus 16.5%) [10], and 30-day mortality (21.4% versus 26.3%) [11] appeared to favor the hypotensive group in two of the studies, of which one was conducted in the prehospital setting and one was performed in the hospital. Additionally, a subgroup analysis found the blunt trauma patients that received controlled resuscitation had decreased mortality compared to those with standard resuscitation (3.2% versus 17.7%; adjusted odds ratio: 0.17; 95% confidence interval: 0.03–0.92) [29]. Overall, it is hard to compare the mortality findings between studies, as each study assessed mortality at slightly different time points.

Secondary outcomes and complications were examined in four of the five randomized trials. In the three prehospital studies, Bickell et al. and Turner et al. found no difference between treatment groups in the rates of respiratory distress syndrome, sepsis, acute renal failure, coagulopathy, wound infection, or pneumonia [7, 8]; Schreiber et al. found no differences in the need for major surgical procedures, renal function, ICU-free days, or ventilator-free days [10]. In Carrick et al.'s in-hospital study, no differences were found in the rate of acute myocardial infarction, stroke, renal failure, hypotension, thrombocytopenia, coagulopathy, anemia, or infection between groups [11]. A longer length of stay [7] and increased acute renal injury [11] were found to be significantly higher in the groups that received more aggressive fluid administration.

It is challenging to determine whether the studies with prehospital interventions or in-hospital interventions produce better outcomes. As shown in Tables 1 and 2, each study within the prehospital time period and in-hospital time period was conducted in different populations and with different interventions. Yet there were also similarities across the groups. Additionally, each randomized controlled trial has limitations. In the Bickell et al. study, very short transportation times were reported from the scene of the injury to the hospital, which may not be generalizable to all geographic locations [7]. The poor compliance by the paramedics to the protocols in the Turner et al. study may have contributed to the fact that no differences in mortality were observed. Also, though both blunt and penetrating traumas were included in the study design, the population consisted predominantly of blunt trauma injuries (98%) [8]. Dutton et al.'s study included all patients, not just those needing to undergo surgery; some of the patients (13.5%) were able to spontaneously undergo hemostasis without needing an operative procedure. Perhaps the injuries to these patients were not severe enough to need hypotensive resuscitation [9]. This limitation is echoed in the Schreiber et al. study who declared that the SBP of 90 mmHg or lower might not be low enough to exclude minimally injured patients since two-thirds of their patients had an Injury Severity Score lower than 15 [10]. Lastly, the randomized controlled trial by Carrick et al. was stopped early due to temporal changes in processes of care, lack of equipoise, slow accrual, and futility and therefore was not powered to see a difference in the primary outcome [11].

## 6. Changing Guidelines

Despite the limitations to each randomized controlled trial, the evidence is pointing towards lower blood pressure and less fluid administration, especially nonblood products. In 2013, the 9th edition of the Advanced Trauma Life Support (ATLS) course made content changes related to resuscitation strategies. The new content

- removed the phrase aggressive resuscitation and now advocates for permissive hypotension before the control of bleeding,
- suggests less crystalloid use (1 L instead of 2) and early use of plasma and platelets in patients that require massive transfusion or in those with significant anticipated blood loss [33].

Similarly, an updated European guideline on the management of bleeding and coagulopathy following major trauma was published in 2013. These recommendations were formulated and graded according to the Grading of Recommendations Assessment, Development, and Evaluation (GRADE) hierarchy of evidence [34]. In this update, there were 37 recommendations covering initial resuscitation and prevention of further bleeding; diagnosis and monitoring of bleeding; tissue oxygenation, fluid, and hypothermia; rapid control of bleeding; and management of bleeding and coagulation. The recommendation specific to hypotensive resuscitation states the following:

- “We recommend a target systolic blood pressure of 80 to 90 mmHg until major bleeding has been stopped in the initial phase following trauma without brain injury.” (Grade 1C)
- “We recommend that a mean arterial pressure ≥80 mmHg be maintained in patients with combined hemorrhagic shock and severe TBI (GCS ≤ 8).” (Grade 1C)

These guidelines also provide a caveat in that patients with TBI and spinal injuries are contraindicated to the hypotensive approach, and careful consideration must be given when treating elderly patients or those with chronic arterial hypertension [35].

## 7. Conclusion

Optimal resuscitation strategies have been examined for nearly a century, more recently with several randomized controlled trials. The randomized controlled trials have demonstrated that aggressive fluid resuscitation in the prehospital and hospital setting leads to more complications than hypotensive resuscitation, with disparate findings on the survival benefit. Recent changes to the ATLS and European guidelines reflect the findings from the randomized controlled trials as well as observational studies and now advocate for lower target blood pressures in trauma patients. Still, since the populations studied in each trial are slightly different, as is the timing of intervention and the targeted vitals, there is still a need for a large, multicenter trial that can examine the benefit of hypotensive resuscitation in both blunt and penetrating trauma patients.

## Figures and Tables

**Table 1:** Prehospital randomized controlled trials' study criteria and outcomes.

Study	Study years	Inclusion criteria	Exclusion criteria	Study arms	Outcomes
Bickell et al. [7]	11/1/1989–12/22/1992	(i) Being ≥16 years (ii) GSW/stab wound to torso (iii) SBP ≤ 90 mmHg	(i) Being pregnant (ii) Revised Trauma Score = 0 (iii) Fatal GSW to head (iv) Minor injuries not requiring surgery	Immediate: rapid infusion of Ringer's solution in-route to hospital Delayed: no fluids in-route to hospital	(i) *Increased survival to hospital discharge in delayed (70%) versus immediate (62%) group* (ii) *Shorter hospital LOS in delayed (11 ± 24 days) versus immediate (14 ± 24 days) groups* (iii) No differences in RDS, sepsis, ARF, coagulopathy, wound infection, or pneumonia

Turner et al. [8]	5/1996–9/1997	(i) Adult trauma patients treated by randomized paramedic crew with at least one of the following: (a) Hospital LOS ≥ 3 days (b) Being admitted to ICU (c) Patients who died after paramedics arrived (d) Being transferred to another hospital (e) Death within 6 months of injury and cause of death was trauma from accident	(i) Being pregnant (ii) Poisonings, hangings, drownings, asphyxiation (iii) Being transported to hospital by helicopter (iv) Being treated by nonrandomized paramedic or doctor (v) Being dead before paramedics arrived at scene (vi) Superficial skin injuries (vii) Any patient with burns (viii) Having the following at admission: isolated fractured neck of femur, single pubic rami fracture, simple facial injury, simple spinal sprain (ix) Patients in “major incidents” (x) Being <16 years (xi) Patients treated by EMT only (xii) Patients referred by GP	Protocol A: fluids started at the scene Protocol B: fluids withheld until hospital arrival	(i) No difference in mortality at 6 months for patients in Protocol A (10.4%) versus Protocol B (9.8%) (ii) No differences in the hospital or ICU LOS (iii) No differences in the proportion of patients with complications

Schreiber et al. [10]	3/2012–4/2013	(i) Blunt or penetrating trauma (ii) Being >15 years (iii) SBP ≤ 90 mmHg (iv) No evidence of severe head injury (v) GCS > 8	(i) Being pregnant (ii) Receiving > 250 mL of fluid before randomization (iii) Receiving CPR by EMS (iv) Drowning (v) Asphyxia due to hanging (vi) Burns on >20% of body (vii) Being incarcerated (viii) >4 hours between call to dispatch and intervention (ix) Ground level falls^*∗*^ (x) Bilateral paralysis^*∗*^	Standard: initial 2 L bolus of fluid with additional fluid to maintain SBP of 110 mmHg Controlled: 250 mL bolus of fluid to maintain SBP of 70 mmHg	(i) No differences in 24-hour mortality (5.2% versus 14.7%) or in-hospital mortality (8.4% versus 16.5%) for the controlled versus standard resuscitation groups, respectively (ii) *Blunt trauma subgroup with controlled resuscitation had decreased mortality (3.2% versus 17.7%)* (iii) No differences in major surgical procedures, renal function, ICU-free days, or ventilator-free days

^*∗*^Exclusions added as the study progressed.

Italicized outcomes are statistically significant, *p* < 0.05.

ARF: acute renal failure; CPR: cardiopulmonary resuscitation; EMS: emergency medical service; EMT: emergency medical technician; GCS: Glasgow coma score; GP: general practitioner; GSW: gunshot wound; ICU: intensive care unit; LOS: length of stay; RDS: respiratory distress syndrome; SBP: systolic blood pressure.

**Table 2:** In-hospital randomized controlled trials' study criteria and outcomes.

Study	Study years	Inclusion criteria	Exclusion criteria	Study arms	Outcomes
Dutton et al. [9]	1996–1999	(i) Arrival at hospital directly from scene (ii) Ongoing hemorrhage (iii) SBP < 90 mmHg in first hour after injury	(i) Being pregnant (ii) Central nervous system injury and altered consciousness or motor function (iii) Being >55 years (iv) History of diabetes or CAD	Conventional: received fluid to a target SBP > 100 mmHg Low: received fluid to a target SBP of 70 mmHg	(i) No difference in in-hospital mortality (7.3% in each group)

Carrick et al. [11]	7/1/2007–3/28/2013	(i) Penetrating trauma (ii) SBP ≤ 90 mmHg (iii) Emergent laparotomy or thoracotomy to control bleeding	(i) Being pregnant (ii) Blunt trauma (iii) Being <14 years or >45 years (iv) Known or suspected head injury (v) Being incarcerated (vi) Those with “opt-out” bracelet	HMAP: targeted intraoperative minimum MAP = 65 mmHg LMAP: targeted intraoperative minimum MAP = 50 mmHg	(i) No differences in 30-day mortality (21.4% versus 26.3%) or 24-hour mortality (13% versus 20%) for LMAP and HMAP groups, respectively (ii) No differences in acute MI, stroke, renal failure, hypotension, coagulopathy, thrombocytopenia, anemia, or infection between groups (iii) *Increased acute renal injury in the HMAP group (30% versus 13%)*

Italicized outcomes are statistically significant, *p* < 0.05.

CAD: coronary artery disease; HMAP: high mean arterial pressure; ICU: intensive care unit; LMAP: low mean arterial pressure; MAP: mean arterial pressure; MI: myocardial infarction; SBP: systolic blood pressure.
